# Performance evaluation of a new on-demand molecular test for the rapid identification of severe acute respiratory syndrome coronavirus 2 in pediatric and adult patients

**DOI:** 10.3389/fmicb.2022.999783

**Published:** 2022-11-03

**Authors:** Luna Colagrossi, Valentino Costabile, Rossana Scutari, Valeria Cento, Luana Coltella, Antonino Reale, Martina Scilipoti, Alberto Villani, Claudia Alteri, Carlo Federico Perno, Cristina Russo

**Affiliations:** ^1^Unit of Diagnostic Microbiology and Immunology and Multimodal Medicine Area, Department of Laboratories, Bambino Gesù Children’s Hospital, IRCCS, Rome, Italy; ^2^Department of Oncology and Hemato-Oncology, University of Milan, Milan, Italy; ^3^Department of Biomedical Sciences, Humanitas University, Milan, Italy; ^4^IRCCS Humanitas Research Hospital, Milan, Italy; ^5^General Pediatrics Unit, Department of Emergency and General Pediatrics, Bambino Gesù Children’s Hospital, IRCCS, Rome, Italy

**Keywords:** SARS-CoV-2, molecular diagnosis, COVID-19, RT-PCR testing, comparative diagnostic accuracy

## Abstract

The rapid spread of severe acute respiratory syndrome coronavirus 2 (SARS-CoV-2) infection has increased the need to identify additional rapid diagnostic tests for an accurate and early diagnosis of infection. Here, we evaluated the diagnostic performance of the cartridge-based reverse transcription polymerase chain reaction (RT-PCR) test STANDARD M10 SARS-CoV-2 (SD Biosensor Inc., Suwon, South Korea), targeting the ORF1ab and E gene of SARS-CoV-2, and which can process up to eight samples in parallel in 60 min. From January 2022 to March 2022, STANDARD™ M10 assay performance was compared with Xpert^®^ Xpress SARS-CoV-2 (Cepheid, Sunnyvale CA) on 616 nasopharyngeal swabs from consecutive pediatric (*N* = 533) and adult (*N* = 83) patients presenting at the “Istituto di Ricovero e Cura a Carattere Scientifico” (IRCCS) Ospedale Pediatrico Bambino Gesù, Roma. The overall performance of STANDARD M10 SARS-CoV-2 was remarkably and consistently comparable to the Xpert^®^ Xpress SARS-CoV-2 with an overall agreement of 98% (604/616 concordant results), and negligible differences in time-to-result (60 min vs. 50 min, respectively). When the Xpert^®^ Xpress SARS-CoV-2 results were considered as the reference, STANDARD™ M10 SARS-CoV-2 had 96.5% sensitivity and 98.4% specificity. STANDARD M10 SARS-CoV-2 can thus be safely included in diagnostic pathways because it rapidly and accurately identifies SARS-CoV-2 present in nasopharyngeal swabs.

## Introduction

Coronavirus disease 2019 (COVID-19) caused by severe acute respiratory syndrome coronavirus 2 (SARS-CoV-2) has been the most significant global outbreak since the Spanish flu of 1918 (Petersen et al., 2020; Burkhard-Koren et al., 2021; Javelle and Raoult, 2021).

Healthcare systems around the world have been challenged by the SARS-CoV-2 pandemic (Wang et al., 2020) and to date, the world counts 6,290,452 deaths from the pandemic (WHO, 2022).

However, the ability of the virus to mutate, as evidenced by a succession of variants of concerns (VOCs), has posed a further challenge to the diagnosis of infection, vaccine development, and clinical treatments (Bian et al., 2021; Chen J. et al., 2021; Chen R. E. et al., 2021; Koyama et al., 2022; McLean et al., 2022).

In the fight against COVID-19, a key role has always been played by diagnosis (WHO Working Group on the Clinical Characterisation and Management of Covid-19 infection, 2020; Peeling et al., 2021). In fact, both at the health facility and community levels, diagnosis of the virus is the first and fundamental step toward its containment. During pandemic, children have been supposed to actively spread SARS-CoV-2 in elderly population and thus many concerns arose about the ability to accurately detect eventual children-related viral variants by PCR methods, previous study demonstrated that genomic SARS-CoV-2 variability was similar across various age categories (Alteri et al., 2022). In line with their findings, we decided to address this aspect by performing an assay comparison in our pediatric clinical setting.

Currently, the field of COVID-19 diagnostic testing is rapidly evolving; diagnostics capable of detecting current infections are antigen- and molecular-based tests. Antigenic tests, which identify viral proteins mostly expressed within nucleocapsid, are far less sensitive but faster than molecular tests, providing the result in about 15–20 min (Dinnes et al., 2021; Pilarowski et al., 2021). However, molecular tests can be performed in batches or as random-access on-demand. Batch tests have high capacities for simultaneous processing (96–200 samples in parallel) but typically have long turn-around-times (4–5 h) (Craney et al., 2020; Gorzalski et al., 2020), favoring their use in cases of large volumes of samples with a low clinical necessity for prompt results (i.e., community screening). Moreover, on-demand tests can process a limited number of samples but provide results in 50 min–2 h (Loeffelholz et al., 2020; Smithgall et al., 2020). Therefore, they are usually dedicated to emergency situations in both community and hospital settings.

Considering the moderate sensitivity of antigenic tests compared to reverse transcription polymerase chain reaction (RT-PCR), the need for molecular tests, which is more rapid and sensitive, fits in.

The STANDARD M10 SARS-CoV-2 (SD Biosensor) is a new cartridge-based, rapid, on-demand RT-PCR test, recently introduced in clinical use, that targets the ORF1ab and E gene of SARS-CoV-2. It runs on the modular STANDARD™ M10 instrument, scalable up to eight modules, and provides results in 20–60 min. Previous studies showed a high diagnostic accuracy (100%) of the STANDARD M10 assay in testing both nasopharyngeal swabs and lower respiratory tract specimens (Colagrossi et al., 2021), with a consistent sensitivity for ≥ 1 gene, the ORF1ab gene, and the E gene (100, 95.5, and 99.5%, respectively) and a full specificity of 100% (Domnich et al., 2022; Ham et al., 2022).

To date, all performance evaluations were achieved on samples from adult individuals. Here, to evaluate the diagnostic performance of this novel assay for the rapid molecular diagnosis of COVID-19 in both pediatric and adult populations, we performed a head-to-head comparison between STANDARD M10 SARS-CoV-2 and the Xpert Xpress SARS-CoV-2, (Cepheid, Sunnyvale, CA), which has analogous similar technical characteristics and is currently recognized as one of the most sensitive tests on the market (Loeffelholz et al., 2020; Smithgall et al., 2020; Serei et al., 2021). Droplet digital PCR (ddPCR), an end-point quantitative PCR method capable of detecting down to a few copies of viral genomes (Alteri et al., 2020; Liu et al., 2020; Suo et al., 2020; Colagrossi et al., 2021), was used to provide additional insights into the ability of rapid cartridge tests to detect up to minimal quantities of SARS-CoV-2 genomes.

## Methods

### Specimen collection and storage

Between January 2022 and March 2022, 616 nasopharyngeal swabs were collected by qualified personnel from consecutive individuals (pediatric patients, their legal tutor or Healthcare Workers) who accessed the Emergency Room of the Ospedale Pediatrico (Rome, Italy) and were immediately transported to the Microbiology Laboratory in 3 mL viral transport medium (VTM) tubes (Copan UTM^®^, Copan, Italy). All samples were tested upon collection with the Xpert Xpress SARS-CoV-2 assay, according to laboratory routine procedure, and with the STANDARD M10 SARS-CoV-2 assay within the next 24 h. In the time between the two tests, samples were conserved at +4°C, according to manufacturer indications. Residual samples were stored at −80°C, and left at the disposal for further analyses, whether deemed necessary.

### STANDARD™ M10 severe acute respiratory syndrome coronavirus 2

The STANDARD™ M10 SARS-CoV-2 cartridge contains PCR buffers and lyophilized real-time RT-PCR reagents. According to the manufacturer’s indications, users should insert 600 μl of sample’s buffer transport medium directly into the cartridge and load it into one module of the STANDARD™ M10 instrument, which allows continuous, random-access loading of up to eight samples. The instrument provides negative or presumptive positive results (amplification of E gene only) automatically in 60 min, while positive results (amplification of both targets) are available in 30–60 min, depending on the viral load. At the end of RT-PCR reaction, the amplification curves and PCR cycle threshold (Ct) values (upper limit of detection, Ct < 40) are released.

### Xpert Xpress severe acute respiratory syndrome coronavirus 2

The Xpert Xpress SARS-CoV-2 is a cartridge-based test that detects the envelope (E) and the nucleocapsid (N2) genomic portions of SARS-CoV-2. The cartridge contains PCR buffers and lyophilized real-time RT-PCR reagents. Users should add 300 μl of sample’s buffer transport medium directly to the cartridge and load it into the GeneXpert instrument, which allows continuous, random-access loading of up to 16 samples. The instrument provides results automatically in 50 min. The results are reported as presumptive positive in the case of identification of the E gene alone, while the detection of N2 always leads to a positive result. At the end of the RT-PCR reaction, the amplification curves and PCR Ct values (upper limit of detection, Ct < 45) are released.

### Severe acute respiratory syndrome coronavirus 2 absolute quantification by Droplet Digital PCR (ddPCR™)

At the end of the study, in case of non-concordant results between the two real-time assays, we exploited samples residuals (conserved at −80°C) to obtain a SARS-CoV-2 RNA absolute quantification by using QX200 Droplet Digital PCR System (Bio-Rad) and targeting RNA-dependent RNA polymerase (RdRp) gene in conserved regions (Alteri et al., 2020). ddPCR targets are amplified and detected using fluorescence-based probes. The fraction of positive droplets is fitted into a Poisson distribution to determine the absolute starting copy number in units of copies/μl of the input sample. We defined a sample as “positive and quantifiable” if at least three droplets were detected.

### Statistical analysis

The performance of diagnostic tools has been compared from both a quantitative and a qualitative point of view. The qualitative comparison was conducted by computing a confusion matrix in order to feed performance descriptive indexes calculation, and Xpert Xpress SARS-CoV-2 system’s results were considered as reference. The differential distribution of false-negative and false-positive responses among system results was also checked with a McNemar’s test under the *null hypothesis* of a different distribution in false response; this test was included because it considers the impact of both false-positive and false-negative response rates simultaneously and returns us a degree of balance between them. Quantitative divergence in Ct’s distribution between systems was statistically evaluated by a paired Student’s *t*-test under the *null hypothesis* that no difference was observed in their mean distribution.

To further assess the overall mean distribution between the two approaches without pairing samples measurement, we relied on the Mann–Whitney test under the *null hypothesis* that divergence was observed among mean distributions.

To further evaluate the overlapping degree between these assays, we performed Bland-Altman test, a popular method to test the agreement between two set of paired measurement which is expressed as a mean bias flanked by an upper and lower limit of agreement.

Moreover, to assess the correlation between age and the detection of SARS-CoV-2 Ct value between the two employed methods, the study population was stratified into five categories according to age: newborns (0–27 days), infants and toddlers (28 days–24 months), children (2–11 years), adolescents (12–17 years), and adults (> 18 years) (Williams et al., 2012).

### Ethical committee

The ethical committee of the Bambino Gesù Children’s Hospital has been notified of the present study. The use of retained clinical specimens for validation of diagnostic methods without individual data and the use of anonymized clinical specimens for validation of diagnostic methods did not require ethical clearance from the ethics committee.

## Results

Between January 2022 and March 2022, we collected and analyzed 616 nasopharyngeal swabs, mainly from pediatric patients (533/616, 86.5%), as we included samples from 27 (4.4%) newborns (0–27 days), 256 (41.6%) infants and toddlers (28 days–24 months), 177 (28.7%) children (2–11 years), and 73 (11.8%) adolescents (12–17 years), in addition to 83 (13.5%) adults (> 18 years).

When Xpress SARS-CoV-2 assay was used as a reference, 115/616 (18.7%) samples provided a positive result with median (IQR) Cts of 19.5 (17.0–23.5), while 501 (81.3%) were negative (Table 1). Confusion matrix reported an overall concordance rate with STANDARD M10 SARS-CoV-2 assay of 98.5% (604/616) (Table 1), consistently maintained across different Ct categories: ≤ 24 (100% concordance; 90/90); 25–29 (100% concordance; 11/11); and ≥ 30 (71.4% concordance; 10/14). STANDARD M10 SARS-CoV-2 assay returned a 96.5% sensitivity and a 98.4% specificity, with a positive predictive value (PPV) of 93.3% (Table 1). False-positive and negative rates have been estimated as being both below 0.05, supported also by McNemar’s chi-squared estimated equal to 0.75 with a *p*-value of 0.386, suggesting that no significant divergence was observed in false response distribution (false positive and false negative) (Table 2).

**TABLE 1:** STANDARD M10 and Xpress SARS-CoV-2: Contingency table.

	M10 Standard			
	
		Positive	Negative	Total
Xpert	Positive	111	4	115
SARS-CoV-2	Negative	8	478	486
	Total	119	482	

**TABLE 2:** STANDARD M10 performance indexes when compared to Xpress SARS-CoV-2 assay used as reference.

M10 performance indexes				
Concordance	98.5(%)			
Sensitivity	96.5(%)			
Specificity	98.4(%)			
Positive predictive value	93.3(%)			
False discovery rate	6.7(%)			
False positive rate	0.16(%)			
False negative rate	0.35(%)			

**Statistical results**			**CI**	
	
Paired Student’s *t*-test H_0_: MeanGroup1 =	t distribution value	5.342	0.508	1.107
MeanGroup2	*P*-value	<0.001		
Paired Student’s *t*-test H_0_: MeanGroup1 =	t distribution value	0.050	0.508	1.107
MeanGroup2 + 0.8Ct	P-value	0.959		
McNemar’s Test	χ^2^	0.750	–	–
H_0_: StandardM10 (FP + FN) Distribution ≠ Xpress(FP + FN) Distribution	*P*-value	0.386	–	–

As reported, sensitivity and specificity are above 95, while the *positive predictive value* (PPV) is slightly lower indicating a true positive call frequency of 93% (within a reasonable precision range). Overall false response rate (FPR, FNR) is consistently below 1%, while the false discovery rate (1-PPV) is around 7%. Paired Student’s *t*-test detects a significant shift in median distribution among assays estimated as being around 0.8; this is confirmed by the loss of significance when the null hypothesis is settled on a true mean of 0.8 Ct. McNemar’s test results inform us that no significant unbalance is observed in the false-positive and false-negative response frequencies.

**FIGURE 1:**
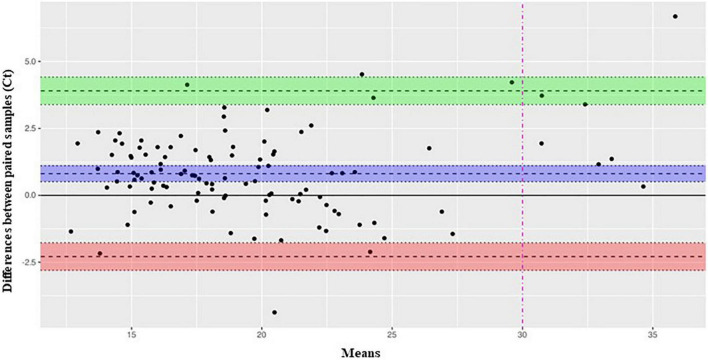
Bland-Altman-plot analysis of SARS-CoV-2 Ct levels measured by Xpress SARS-CoV-2 and STANDARD MIO SARS-CoV-2 assays. Bland-Altman plot analysis of 109 positive samples tested with both assays. On y-axis are reported the differences between paired samples while on x-axis their means. Limit of agreement (+1.96 SD) is represented by a green **(upper limit)** and red **(lower limit)** bands. The midline bend **(blue)** represent the mean bias observed between the assay estimated around 0.8 Ct.

Median (IQR) Ct values of the 111 concordant positive samples were comparable between the two assays [19.4 (17.0–22.8) with Xpress SARS-CoV-2 vs. 18.7 (15.9–22.6) with STANDARD M10 SARS-CoV-2; *p* = 0.106], and Student’s *t*-test indicates a difference in mean distribution estimated around 0.8 Ct. This correspondence was preserved across all age groups analyzed, from newborns to adults, with no significant difference detected (Table 3). As for previous statistical test applied in this study, this result has been also confirmed by Bland Altman test (Figure 1), showing an average bias between two assays estimated around 0.8 Ct; under a clinical point of view this value slightly impact the discrimination between a positive and negative sample. Furthermore, above the clinical threshold of 30 Ct we observed a good degree of concordance given that most of the samples lay in proximity of median difference (blue bend) while only two (along green band, in range 30–32 Ct) present a positive difference in mean that could bring their values outside the limit of detection. Below this threshold we observe the most of the samples lying in the range 10–25 Ct, in this range the extent of limit of agreement (−2.2862–3.9015) slightly impact diagnostic decision given that, in worst case, a positive difference of 4 Ct would maintain the sample inside the Limit of detection for positivity.

**TABLE 3:** SARS-CoV-2 Ct distribution over 111 true positive samples among the five age groups.

Study population	N (%)	Range of age, median (IQR)	Xpert SARS-CoV-2 CT in positive samples, median (IQR)	M10 SARS-CoV-2 CT in positive samples, median (IQR)	*P*-value CT
Overall	111	0.7 (0.3–8) years	19.4 (17.0–22.8)	18.7 (15.9–22.6)	0.106
Newborns (0–27 days)	8 (7.2)	23 (16–26) days	18.7 (16.8–21.0)	18.3 (16.0–20.8)	0.721
Infants and toddlers (28 days to 24 months)	55 (49.5)	4 (2–6) months	18.4 (16.4–21.1)	17.0 (15.3–21.0)	0.117
Chidren (2–11 years)	26 (23.4)	5 (3–8) years	19.4 (17.8–23.2)	18.5 (16.8–23.4)	0.464
Adolescents (12–17 years)	13 (11.7)	13 (12–15) years	21.8 (19.4–23.6)	21.0 (19.5–22.8)	0.511
Adults (>18 years)	9 (8.1)	39 (36–44) years	22.5 (21.2–24.0)	23.0 (19.1–23.4)	0.863

**P-value for statistical median comparison in reported sub groups has been obtained with Mann-Whitney test.

**FIGURE 2:**
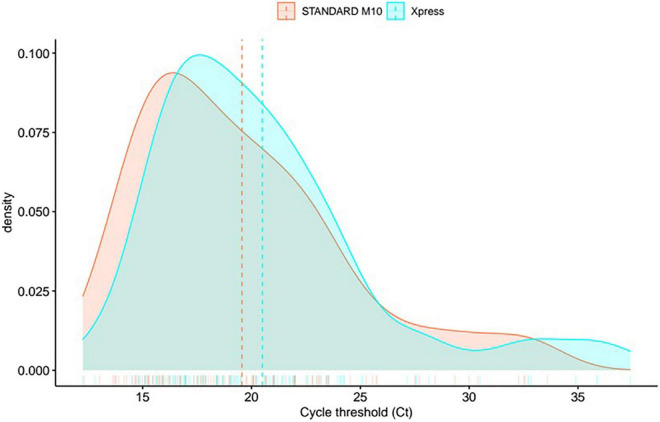
Density distribution in mean gene Ct values between two systems (mean between gene E and N for Xpress SARS-CoV-2, gene E and Orflab for STANDARD M10 SARS-CoV-2).

The density distribution of mean Ct values confirmed no consistent variation between the two assays (Figure 2). We observed decay in signal density around 30 Ct for Xpress SARS-CoV-2 assay, while STANDARD M10 retained a flat trend; further to this point, the signal of the former shows a longer tail, spanning up to ∼40 Ct, while the latter shows a rapid decay of around 35 Ct. These results suggest that, while Xpress SARS-CoV-2 assay shows a higher limit of detection, STANDARD M10 presents a more linear performance of around 30 Ct.

**FIGURE 3:**
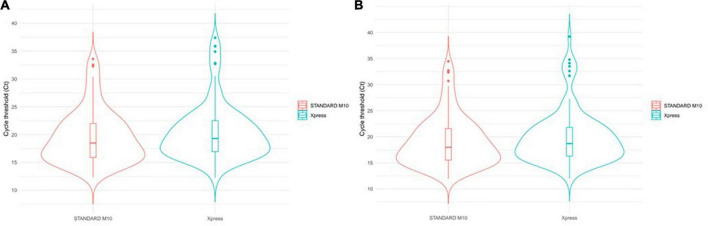
SARS-CoV-2 Ct distribution comparison. Violin width informs about the number of elements observed at any Ct level. Upper dots can be considered as outliers since far from the mam population. Internal boxplots provide a dimension of IQR and median values. Among samples tested with two systems, violin plots compare: **(A)** Mean gene Ct value distribution; **(B)** E gene Ct value distribution.

To visualize mean gene Ct distribution among samples, we exploited the violin plots (Figure 3). This kind of chart provides double information by simultaneously reporting Ct distribution for both assays and population density represented by violin width.

By visually inspecting this chart, we observed that the highest population density for both methods, represented by the largest level of the violin, overlaps each other. This is also confirmed by the internal boxplot plot, whose extent is highly comparable (Figure 3), and by the Mann–Whitney test, which returned a not significant *p*-value of 0.239.

For 11 out of the 12 discordant results, the volume of the residual sample was sufficient for absolute SARS-CoV-2 load quantification by ddPCR. This further investigation allowed us to detect SARS-CoV-2 genetic material in 2 out of 7 (28.6%) of the Xpress negative/STANDARD M10 positive (false positive) samples, suggesting that STANDARD M10 actually provided a true positive result. Similarly, ddPCR indicated the absence of SARS-CoV-2 genetic material in 2 out of 4 (50%) Xpress positive/STANDARD M10 negative (false negative) samples, suggesting that STANDARD M10 actually provided a true negative result (Table 4 and Supplementary Table 1).

**TABLE 4:** Comparison of discordant results among the three methods.

	Xpress SARS-CoV-2,		M10 Standard,		ddPCR,	M10 standard result	
	Cepheid		ReLab		Bio-Rad	interpretation	
				
	Gene N, Ct	Gene E, Ct	Gene Orf1ab, Ct	Gene E, Ct	Gene RdRp, copies/mL	Xpress SARS-CoV-2 as reference	ddPCR as reference
1	TND	TND	TND	35.38	TND	False positive	False positive
2	TND	TND	TND	36.1	TND	False positive	False positive
3	TND	TND	TND	35.46	TND	False positive	False positive
4	TND	TND	TND	34.85	TND	False positive	False positive
5	TND	TND	34.14	35.58	207	False positive	**True positive**
6	TND	TND	35.2	TND	TND	False positive	False positive
7	TND	TND	34.12	TND	65	False positive	**True positive**
8	40.5	TND	TND	TND	242	False negative	False negative
9	39.2	TND	TND	TND	TND	False negative	**True negative**
10	TND	40.1	TND	TND	TND	False negative	**True negative**
11	38.6	TND	TND	TND	1028	False negative	False negative

TND, Target Not Detected.

## Discussion

With rapid antigenic tests dominating the scenario of home-base and community SARS-CoV-2 testing, the mandatory features that we require from a hospital-based RT-PCR assay working aside from emergency departments should be (a) rapid, (b) easy to perform (with minimal on-hand time), (c) very reliable, and (d) relatively cheap in order to provide the prompt indications needed for critical patient management.

This study is the first study aimed to evaluate the performance of the RT-PCR STANDARD™ M10 for the rapid molecular diagnosis of SARS-CoV-2 in nasopharyngeal swabs from pediatric patients. The STANDARD™ M10 SARS-CoV-2 (SD Biosensor) is a newly available rapid, on-demand RT-PCR assay intended to detect the envelope and ORF1ab genomic portions of SARS-CoV-2 from nasopharyngeal swabs. It has an on-hand time of 1 min and a total per-sample turnaround time of 20–60 min. It runs on a completely automated, dedicated instrument that can process up to eight samples in parallel. According to manufacturers, its sensibility and specificity in detecting SARS-CoV-2 in samples from adult individuals is 100%. Yet, no real-life study has reported its clinical use so far, and no data are available on its performance in the pediatric population.

As a result, we conducted a diagnostic performance study on 616 specimens collected from 533 children [of whom 309 (58%) were under the age of 24 months] and 83 adults who visited the Emergency Department at Bambino Gesù Children’s Hospital. As a reference, we used the Xpert^®^ Xpress SARS-CoV-2 assay (Cepheid), currently used in our routine screening protocols at hospital admission. This comparison was deemed to be highly clinically significant, as the two tests share all the main technical features that are required for a rapid hospital-based ER RT-PCR assay, including: (a) continuous random-access loading of disposable cartridges; (b) poor hands-on time; (c) fully automated walk-away processing with a per-sample turn-around-time of less than 1 h; (d) simultaneous detection of two genomic targets; and (e) a compact architecture that fits even in the smallest laboratories. In addition, the Xpert^®^ Xpress SARS-CoV-2 assay is one of the most sensitive tests on the market (Loeffelholz et al., 2020; Smithgall et al., 2020; Serei et al., 2021) and is currently recognized as a benchmark in ER settings, making our comparison particularly challenging. Yet, our performance indexes computed by confusion matrix highlighted a 96.5% sensitivity and a 98.4% specificity for the STANDARD™ M10 SARS-CoV-2, with a very limited number of discordant results (*n*/*N* = 12/616), mainly associated with an increased rate of positive results by STANDARD™ M10 assay (putative false-positive, *n*/*N* = 8/12 discordances). Worth of note, all of 12 analyzed discordant samples were characterized by a Ct value greater than 34 and a positivity to only one target gene (as shown in Table 4). We do not expect bias introduced by samples management since we strictly followed CDC guidelines (CDC, 2022) and by literature inspection we found well conducted study showing that +4°C or −20°C swabs storage does not significantly affect feasibility of RT-PCR tests for SARS-CoV-2 (Veguilla et al., 2022). Notably, when ddPCR was used to further assess SARS-CoV-2 presence in discordant samples, 2 out of 7 (28.6%) of the putative false-positive results were actually reclassified in true-positives, and 2 out of 4 (50.0%) of the putative false-negative results were reclassified in true-negative. This suggests that the diagnostic performance of STANDARD™ M10 assay in our clinical setting is reasonably expected to be non-inferior to that of the Xpert^®^ Xpress SARS-CoV-2 assay, both in terms of sensitivity and specificity.

The Ct values at RT-PCR are commonly used as a proxy for viral-load quantification (Walker et al., 2021), even if a non-optimal correspondence has been highlighted (Rao et al., 2020). Yet, in clinical practice, Cts are a rapid and easy way to “approximate” viral content in clinical samples and are a useful parameter that contributes to the cross-sectional and multidisciplinary clinical evaluation that takes place at any ER entrance. Our study provided reassuring results on the consistency of Ct values between the two assays, with no significant differences in mean values distribution and limited divergence, always < 1 log. We need to underline that the Ct values cannot be directly compared between assays of different types due to the chemistry of reagents, gene targets, cycle parameters, analytical interpretive methods, sample preparation and extraction techniques, and inherent randomness and variation in the sensitivity of the method (Falasca et al., 2020). Yet, the differences we found (or, better, the lack thereof) support the hypothesis that the use of this test will not significantly infer to patient’s management outcome from a clinical perspective compared to a high-quality standard test, such as Xpert^®^ Xpress SARS-CoV-2 assay.

Our study may have potential limitations. Not all 616 samples have been tested by ddPCR and, therefore, no full performance comparisons with this highly sensitive assay could be made. Yet, the commercial RT-PCR test we used as a reference is the current benchmark for clinical SARS-CoV-2 diagnosis in emergency settings, ensuring the clinical significance of our study in the context of commercially available, CE-marked assays. In addition, SARS-CoV-2 variants have not been routinely tested in the analyzed samples, yet (a) all samples were collected in the period from January to March 2022, when SARS-CoV-2 infections in Italy were almost entirely attributed to Omicron VOC (Rapporto, 2022), and (b) we sequenced a remarkable number of samples in that period and confirmed this rate of variants (Alteri et al., 2022).

In conclusion, our results support the optimal diagnostic performance of the novel STANDARD™ M10 SARS-CoV-2 assay in a real-life ER setting, constituted by pediatric patients in comparison with adult patients. The remarkable degree of similarity in terms of reliability, sensitivity, and specificity, with our current diagnostic reference assay, supports the implementation of STANDARD™ M10 SARS-CoV-2 in a modern diagnostic approach to SARS-CoV-2 infection. On this basis, the availability of a molecular test, fast, easy, and reliable becomes of major importance in the management of patients that require a molecular test (i.e., emergency rooms, fragile patients, etc.), particularly if it is less expensive and therefore sustainable, considering also the decrease of the budget in many countries dedicated to the fight against COVID-19.

## Data availability statement

The raw data supporting the conclusions of this article will be made available by the authors, without undue reservation.

## Ethics statement

Ethical review and approval was not required for the study on human participants in accordance with the local legislation and institutional requirements. Written informed consent from the participants’ legal guardian/next of kin was not required to participate in this study in accordance with the national legislation and the institutional requirements.

## Author contributions

LC, RS, and LuaC: performed the experiments. LC, VCo, and CR: designed the study and wrote the manuscript. AR, MS, and AV: contributed to the samples collection. LC, VCo, and RS: processed the data. LC and VCo: data curation and editing. CP, CR, VCe, and CA: revised data. CP and CR: supervision. All authors contributed to the article and approved the submitted version.
