# Supplementary material for: Performance evaluation of a new on-demand molecular test for the rapid identification of severe acute respiratory syndrome coronavirus 2 in pediatric and adult patients
Source: Front Microbiol. 2022 Nov 3;13:999783. doi: 10.3389/fmicb.2022.999783 (PMC9670180; doi:10.3389/fmicb.2022.999783)
Supplement: Supplementary file 1 [file Table_1.pdf]

Table S1. STANDARD M10 performance indexes when compared to Xpress SARS-CoV-2 assay used as reference and corrected by ddPCR for discordant results

M10 Standard			
	**Positive**	**Negative**	**Total**
**Positive**	113	2	115
**Negative**	6	480	486
**Total**	119	482	
**M10 Performance indexes**			
	**Estimated point (%)**	**Lower C.I.**	**Upper C.I.**
**Concordance**	98.7%		
**Sensitivity**	94.9%	94.8%	95%
**Specificity**	99.5%	99.5%	99.6%
**Positive Predictive Value**	98.2%	98.1%	98.3%
**Negative Predictive Value**	98.8%	98.7%	98.8%
**False Discovery Rate**	5.0%		
**False Positive rate**	1.2%		
**False Negative rate**	1.7%		

The integration of results obtained by ddPCR led to the re-assignment of 4 outcome; in particular 2 False Positive sample have been detected as being True positive while 2 False negative sample have been re-assigned as True positive. Thus the overall score for sensitivity and specificity has improved as well as positive/negative predictive values and false rate.
